# Supplementary figures and images for: Urobiome Signatures of Recurrent Urinary Tract Infections in Adolescent Pregnancy: A Longitudinal Study
Source: Microorganisms. 2025 Oct 21;13(10):2406. doi: 10.3390/microorganisms13102406 (PMC12566430; doi:10.3390/microorganisms13102406)

*Escherichia coli*

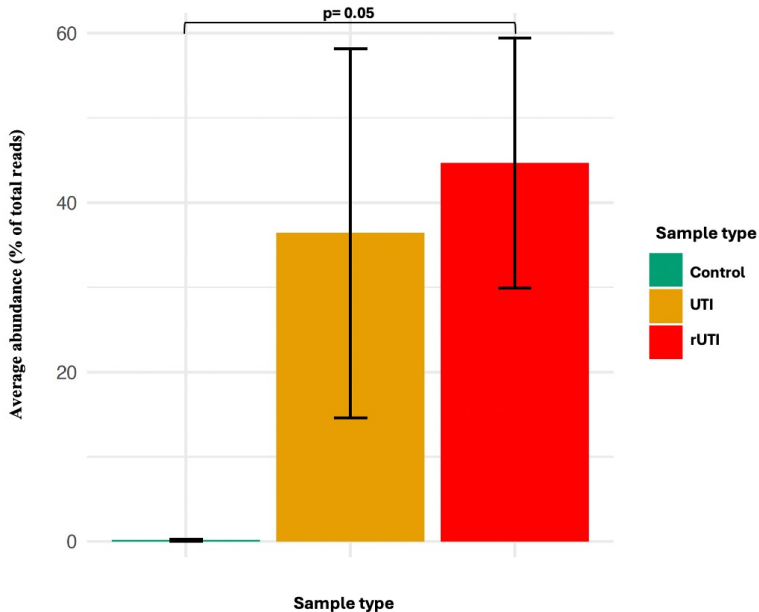

Supplement: Supplementary file 1 [file microorganisms-13-02406-s001.zip › Figure S1.pdf]

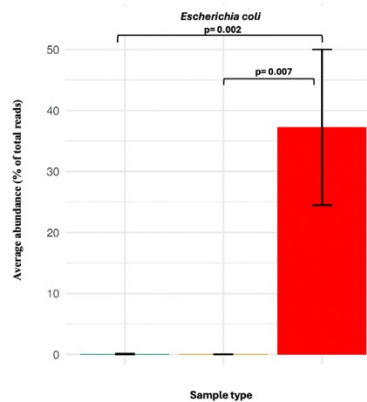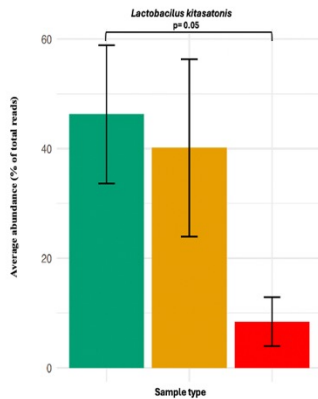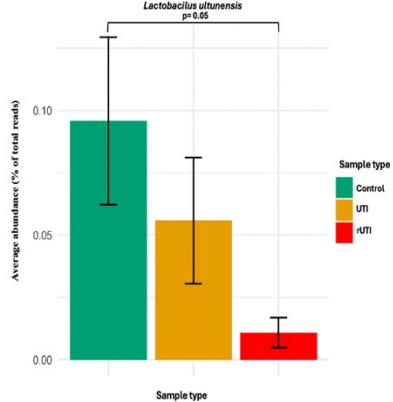

Supplement: Supplementary file 1 [file microorganisms-13-02406-s001.zip › Figure S2.pdf]

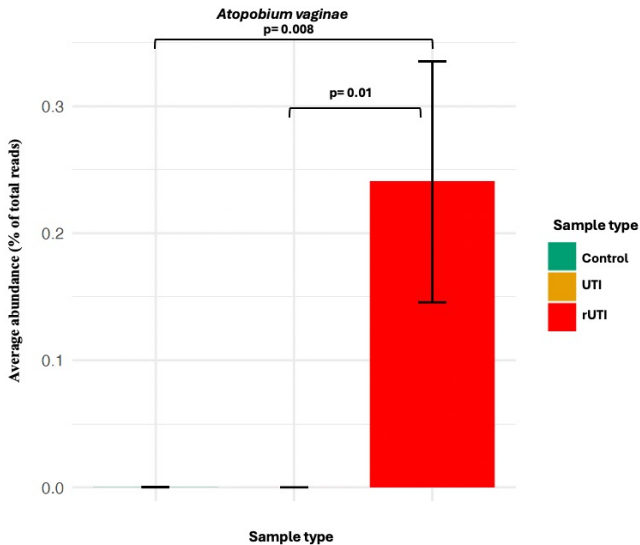

Supplement: Supplementary file 1 [file microorganisms-13-02406-s001.zip › Figure S3.pdf]

PCA using canberra distance

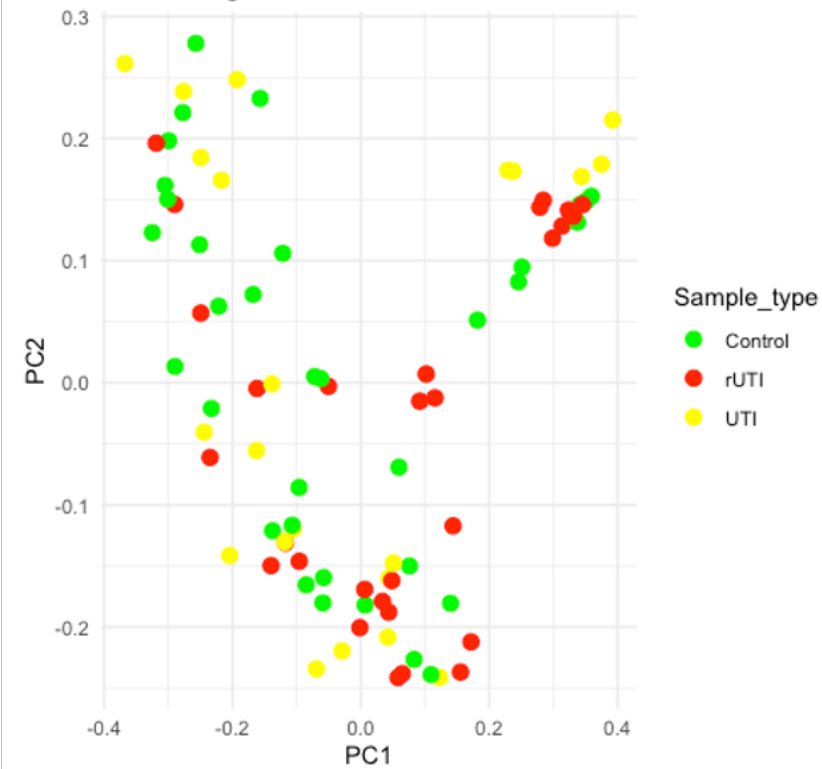

PCA using jaccard distance

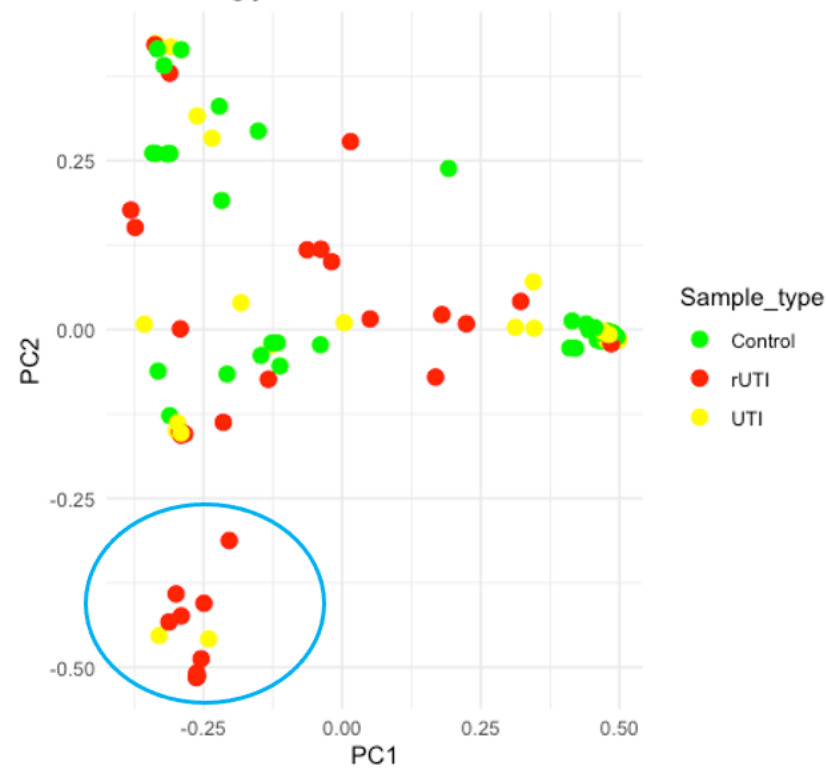

PCA using bray distance

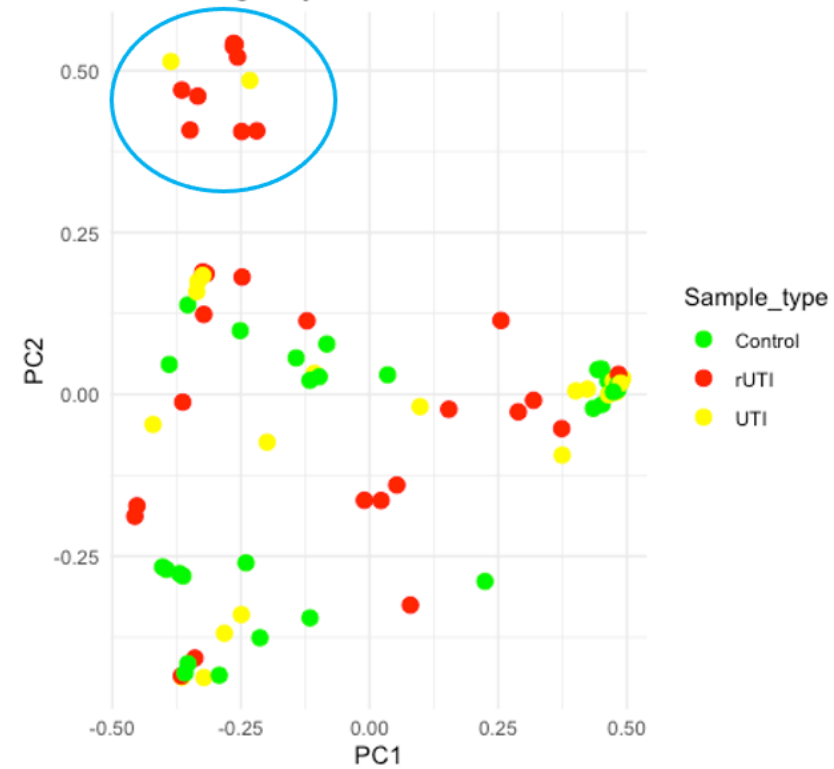

Supplement: Supplementary file 1 [file microorganisms-13-02406-s001.zip › Figure S4.pdf]
